# The Impact of Educational LLM Agent Use on Teachers’ Curriculum Content Creation: The Chain Mediating Role of School Support and Teacher Self-Efficacy

**DOI:** 10.3390/bs16010124

**Published:** 2026-01-15

**Authors:** Huifen Xu, Minjing Chen, Minjuan Wang, Jijian Lu

**Affiliations:** 1 China Education Modernization Research Institute, Hangzhou Normal University, No. 2318 Yuhang Tang Road, Yuhang District, Hangzhou 311121, China; nie24.xh0449@e.ntu.edu.sg; 2 Zhejiang Philosophy and Social Science Laboratory for Research in Early Development and Childcare, Hangzhou Normal University, No. 2318 Yuhang Tang Road, Yuhang District, Hangzhou 311121, China; 3 National Institute of Education, Nanyang Technological University, 1 Nanyang Walk, Singapore 637616, Singapore; 4 Jing Hengyi School of Education, Hangzhou Normal University, No. 2318 Yuhang Tang Road, Yuhang District, Hangzhou 311121, China; 2025111004019@stu.hznu.edu.cn; 5 Global Institute for Emerging Technology, The Education University of Hong Kong, Hong Kong, China; 6 School of Social Sciences, Nanyang Technological University, 1 Nanyang Walk, Singapore 637616, Singapore

**Keywords:** educational LLM agents, teacher content creation, curriculum design, school support, self-efficacy

## Abstract

The application of social cognitive theory has expanded to the boundaries of human-computer interaction research. However, existing research has scarcely addressed mutual cognitive facilitation between humans and personalized educational large language model (LLM) agents. This study explored how educational LLM agents influence teachers’ curriculum design and content creation, based on a sample of 464 teachers from coastal regions of China, along with semi-structured interviews with 23 participants. Quantitative analysis of the survey data revealed that the involvement of educational LLM agents positively predicts teachers’ ability to create content in curriculum design. Additionally, teachers’ self-efficacy mediated this relationship, while both school support and self-efficacy together created a chain mediation effect. Qualitative findings from the interviews supported the quantitative results and further highlighted individual differences and contextual nuances in teachers’ use of educational LLM agents. In summary, the findings indicated that educational LLM agents positively impact teachers’ curriculum design and content creation, with school support and teachers’ self-efficacy acting as a chain mediator in this process.

## 1. Introduction

Educational large language model (LLM) agents refer to applications developed based on Generative Artificial Intelligence (GenAI). They can automatically generate teaching resources such as texts, images, audios, and videos in educational scenarios (Cheung, 2025), participate in the teaching process with educational interactive roles, adapt to needs through teacher–student interactions, provide interactive support for teachers in curriculum content creation, and have become an important way to integrate AI technology into the field of education. By providing personalized feedback and incorporating interdisciplinary resources, educational agents create new opportunities for teachers in curriculum design (Dever et al., 2024; Stevenson et al., 2024). Since curriculum design is a fundamental aspect of teachers’ professional practice, the quality of content creation directly influences instructional effectiveness and student learning experiences. The introduction of educational LLM agents not only transforms the technological framework underlying curriculum development but also imposes new demands on teachers’ cognitive and behavioral patterns (Bautista et al., 2021). Previous studies indicated that educational LLM agents can enhance teachers’ engagement in curriculum design by improving resource integration efficiency and facilitating collaboration (Zhai, 2025). However, concerns persist regarding low levels of technology acceptance and anxiety associated with their use (Granström & Oppi, 2025). A particularly important factor is teachers’ perception of their own competence—self-efficacy. Teachers with high self-efficacy are more likely to explore the potential of educational LLM agents and translate these opportunities into curriculum innovation (Li & Zeng, 2025). At the institutional level, school support, including training and policy incentives, serves as a critical contextual variable that can directly mitigate the challenges of adopting educational LLM agents and, indirectly, enhance teachers’ content creation through improved self-efficacy (Shen et al., 2024).

Despite these insights, some significant gaps remain. First, most research has concentrated on the impact of educational LLM agents on student learning (Sharma et al., 2025; Lim et al., 2025), leaving the mechanisms that shape teachers’ curriculum design practices underexplored. Second, the interaction between school support and teachers’ self-efficacy as mediators in the relationship between educational LLM agent use and content creation is not well understood, particularly regarding the potential for a chain mediation pathway. These gaps hinder our understanding of how educational LLM agents can effectively empower teachers’ curriculum innovation. Finally, current research on secondary school teachers driven by AI education policies remains scarce, with existing studies predominantly quantitative in nature (Ferikoğlu & Akgün, 2022), rendering it difficult to address deeper issues concerning teachers’ subjective perceptions and organizational dynamics. This study employed a mixed-methods approach, utilizing quantitative surveys to capture macro-level characteristics while employing qualitative interviews to explore micro-level experiences and organizational logic. This targeted methodology addresses existing research gaps, highlighting dual innovation in both contextual relevance and methodological design.

Guided by social cognitive theory (Bandura, 1986), this study aimed to examine the influence of educational LLM agents on teachers’ curriculum content creation, with a particular focus on the mediating roles of school support and self-efficacy. Specifically, the study addressed the following questions:

1. To what extent and in what ways does the application of educational LLM agents exert a significant influence on the quality of teachers’ curriculum content creation?
2. What is the mediating role and effect size of school support in the association between educational LLM agents utilization and teachers’ curriculum content creation quality?
3. To what degree does teachers’ self-efficacy mediate the relationship between the use of educational LLM agents and the quality of their curriculum content creation?
4. How does school support indirectly boost the quality of teachers’ curriculum content creation by improving their self-efficacy, and what is the magnitude of this chain mediation effect?

By elucidating these mechanisms, this study contributed to the theoretical understanding of the intersection between educational technology and teacher professional development, while also providing practical implications for schools aiming to promote the adoption of educational LLM agents and enhance teachers’ capacity for curriculum innovation.

## 2. Literature Review

### 2.1. The Impact of Educational LLM Agents on Teachers’ Curriculum Content Creation

Curriculum content creation refers to a series of organized activities guided by teaching objectives, centered around online teaching resources, knowledge content systems, and the pre-class, in-class, and post-class teaching phases (Liang & Lu, 2025). Educational LLM agents, defined as interactive tools that integrate artificial intelligence technologies (Yaseen et al., 2025), influence teachers’ curriculum content creation through features such as intelligent support, immersive virtual reality, and intelligent tutoring systems (Zawacki-Richter et al., 2019), and their application value in curriculum content creation has been initially verified by existing studies.

From a positive impact perspective, the technical advantages of educational LLM agents provide multiple boosts for curriculum content development. On one hand, these agents can expand teachers’ perspectives in content creation by integrating interdisciplinary resources (Norton et al., 2022) and simulating teaching scenarios (Theeuwes et al., 2025), thereby enhancing instructional efficiency. On the other hand, tools like Khan Migo and Duolingo enable teachers to provide more personalized instruction (Pandey & Singh, 2025). Moreover, educational LLM agents assist teachers in identifying their pedagogical strengths and optimizing content design (Zangana et al., 2025). By delegating repetitive and routine tasks to AI-driven systems, teachers can concentrate on higher-order activities, such as fostering critical thinking and engaging in curriculum innovation (Kayal, 2024). Similarly, Lu et al. (2022) demonstrated that integrating adaptive learning technologies into mixed-reality environments can enhance pre-service teachers’ creativity in expanding instructional scenarios. In short, teachers’ curriculum content creation is closely related to educational LLM agents. Research findings indicate that the use of educational LLM agents plays a key role in enhancing teachers’ ability in curriculum content creation.

### 2.2. The Mediating Role of School Support

School support (such as resource allocation and institutional incentives) acts as a critical organizational variable, providing the contextual foundation for effectively applying educational agents and supporting teachers’ curriculum content creation (Cai & Tang, 2021). Specifically, adequate support and access to resources enable more effective integration of artificial intelligence into teachers’ instructional practices and curricular innovation (Molefi et al., 2024). Within China’s educational sphere, the range of free educational LLM agents and database resources available to teachers continues to expand. These resources differ from the paid databases and tools procured by higher education institutions in terms of their service positioning and accessibility thresholds. By leveraging their convenience and practicality, these free educational LLM agents have effectively enhanced core teaching experiences such as lesson preparation and research support, garnering substantial positive user feedback. This has driven their adoption at the institutional level, prompting universities to proactively provide complementary measures, including resource integration, technical adaptation, and training support.

Empirical studies suggested that when teachers perceive strong school support, their motivation increases, and this strengthens their willingness to engage in educational innovation (Lam et al., 2010). Moreover, organizational recognition from schools can enhance teachers’ innovative capacity (Xiang et al., 2024). Supportive environments, professional development resources, and leadership support also contribute positively to teachers’ curriculum content creation (Cai & Tang, 2022). Therefore, school support has the potential to improve the level of curriculum content creation of teachers.

### 2.3. The Mediating Role of Teacher Self-Efficacy

Teacher self-efficacy (Bandura, 1997), defined as teachers’ belief in their ability to successfully complete curriculum design tasks, serves as a crucial psychological bridge connecting educational LLM agents to curriculum content creation. Using artificial intelligence can boost teachers’ self-efficacy, stimulate their creativity, and, in turn, support their professional development (Lu et al., 2024).

Research showed that improving teachers’ digital literacy, including digital knowledge and digital technology application, can enhance their self-efficacy (Yao & Wang, 2024). Higher self-efficacy, in turn, encourages teachers to persist in innovation, thereby boosting creativity in curriculum content creation (Bas, 2021). Conversely, low self-efficacy may lead teachers to over-rely on pre-set outputs from educational agents, which constrains originality and authentic expression (Xu et al., 2025). In this way, we anticipated that the application of educational LLM agents may enhance teachers’ content creation capacity by boosting their self-efficacy.

### 2.4. The Chain Mediating Role of School Support and Teacher Self-Efficacy

School support and teacher self-efficacy do not operate in isolation; rather, they may interact to form a chain mediation pathway in the relationship between educational LLM agent use and curriculum content creation. Prior research has found that while school support directly improves the quality of teachers’ content creation, it also provides the confidence and motivation teachers need to apply educational concepts and integrate instructional resources (Liang & Lu, 2025).

On the one hand, enhanced school support, including collegial support, leadership encouragement, and an inclusive school climate, can significantly strengthen teachers’ self-efficacy (Shirli et al., 2022). On the other hand, when teachers with high self-efficacy work in supportive school contexts, they are more likely to turn technological tools into creative advantages, thereby increasing their professional engagement. In this sense, school support provides external conditions for cultivating self-efficacy, while self-efficacy transforms external support into intrinsic creative motivation.

Empirical evidence further showed that organizational support (e.g., training and resources) amplifies the positive impact of teacher–AI collaboration on self-efficacy, while higher technological self-efficacy enhances teachers’ agency and instructional innovation (Ding et al., 2025). It can be inferred that there is a significant correlation between school support and teachers’ self-efficacy, and the application of educational LLM agents may indirectly influence teachers’ curriculum content creation through school support and teachers’ self-efficacy.

## 3. The Research Hypotheses

This study indicated that the application of educational LLM agents may affect teachers’ curriculum content creation. However, the impact of educational LLM agent applications on curriculum content creation may not be direct; it could also be mediated by school support and teachers’ self-efficacy. Furthermore, school support may mediate the influence of teachers’ self-efficacy on curriculum content creation. Therefore, the mediating effect of educational LLM agent applications on curriculum content creation may depend on the level of school support and teachers’ self-efficacy.

**Figure 1:**
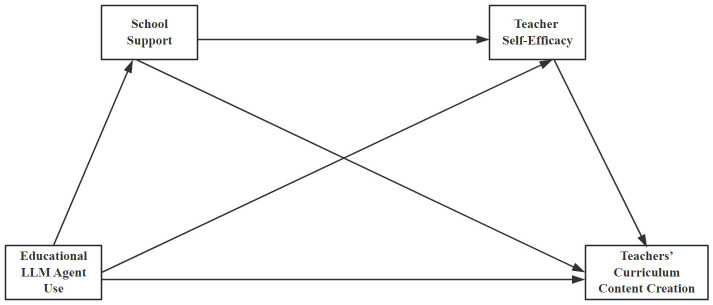
Hypothesized Research Model.

In summary, the following hypotheses (see Figure 1) are proposed.

**Hypothesis 1** **(H1).** *The application of educational LLM agents positively predicts teachers’ curriculum content creation.*

**Hypothesis 2** **(H2).** *School support mediates the relationship between educational LLM agent use and teachers’ curriculum content creation.*

**Hypothesis 3** **(H3).** *Teacher self-efficacy mediates the relationship between educational LLM agent use and teachers’ curriculum content creation.*

**Hypothesis 4** **(H4).** *School support and teacher self-efficacy jointly form a chain mediation pathway between educational LLM agent use and curriculum content creation.*

## 4. Method

### 4.1. Participants

To ensure the reliability and validity of the survey instrument, we conducted a pilot study before formal data collection. Based on the pilot results and participants’ feedback, we revised the questionnaire items to ensure clarity and accuracy of expression. During the formal phase, we distributed the questionnaire online via Wen Juan Xing (Questionnaire Star). We collected a total of 520 questionnaires. After excluding 56 invalid ones (including those with missing data, blank items, or duplicate options), we finally obtained 464 valid questionnaires, with an effective recovery rate of 89.2%.

Based on educational LLM agents usage, the final sample included 464 teachers from economically developed coastal regions of China. It should be clarified that the term ‘coastal developed regions’ as defined in this study specifically refers to coastal provinces such as Zhejiang, Fujian, and Jiangsu, which show relatively high levels of economic development. Although these provinces rank among the nation’s leaders in overall socio-economic advancement, they still contain extensive rural areas, demonstrating a pronounced urban-rural development gradient. During the data collection phase, questionnaires were distributed through teacher training programmes. These training sessions encompassed not only urban school teachers but also a substantial number of frontline educators from rural areas within the aforementioned developed provinces. The demographic characteristics of the participants were as follows: By gender, female teachers formed the majority (*n* = 315, 67.9%), reflecting the gender distribution of teachers in China’s basic education sector. By household registration, rural-registered teachers accounted for the majority (*n* = 350, 75.4%), which aligns with the context of data collection. However, the sample also included 114 teachers with urban household registration, ensuring a degree of representativeness. By teaching grade, most participants taught Grade 7 (*n* = 215, 46.4%) and Grade 8 (*n* = 147, 31.7%); this distribution may relate to lower secondary teachers’ higher willingness to participate in the survey. By teaching subject, science (*n* = 188, 40.5%) and mathematics (*n* = 163, 35.1%) teachers were the most common, while information technology teachers were relatively few. This distribution matches subject allocations in Chinese lower secondary schools. Importantly, these subjects are closely linked to the application of educational agents in teaching practice, making the sample may be employed to investigate the effects of educational agent use in STEM-related disciplines. Detailed demographic information is provided in Table 1.

### 4.2. Instruments

#### 4.2.1. Key Scales

##### Educational LLM Agent Use Scale

We adapted the Educational LLM Agent Use Scale from the Human-Centered Artificial Intelligence Application Scales, developed by Berretta et al. (2023) and Jaboob et al. (2024), to align with the application of educational LLM agents in curriculum content creation (see Appendix A). This adapted scale includes four items, such as “educational LLM agents will improve my teaching practices and interaction with digital resources”, each rated on a five-point Likert scale. It demonstrated high internal consistency, with a Cronbach’s alpha coefficient of 0.943, confirming strong reliability and validity.

##### School Support Scale

We adapted the School Support Scale from the “Guidance” subscale of Arnold et al.’s (2000) questionnaire and the “Skill Development” subscale of Konczak et al. (2000), tailoring it to the context of school-level support for educational LLM agent use by teachers and students (see Appendix A). This adapted scale includes four items, such as “Both the institution and teaching staff provide support regarding the utilization of educational LLM agents”, all measured on a five-point Likert scale. With Cronbach’s alpha coefficient of 0.924, it demonstrated strong internal consistency and validity.

##### Teacher Self-Efficacy Scale

We adapted the Teacher Self-Efficacy Scale from the General Self-Efficacy Scale, developed by Schwarzer and Jerusalem (1995), to contextualize it around teachers’ use of educational LLM agents in curriculum content creation (see Appendix A). This adapted version includes five items, such as “I possess confidence in executing course design content creation effectively”, each measured on a five-point Likert scale. With Cronbach’s alpha coefficient of 0.954, it confirmed high reliability and validity.

##### Curriculum Content Creation Scale

We developed the Curriculum Content Creation Scale (see Appendix A) with reference to Jaboob et al. (2024). This scale includes four items, such as “I believe educational LLM agents will positively impact my curriculum design capabilities and teaching content creation”, each rated on a five-point Likert scale. With Cronbach’s alpha coefficient of 0.956, it further demonstrates high reliability and validity.

However, the Cronbach’s α coefficients for all four scales in this study were ≥0.9, suggesting potential overlap in item content. Therefore, to exclude the potential impact of excessively high α values on validity, we conducted further structural validity tests, with all indicators meeting the criteria. Firstly, overall model fit for the four-factor structure: Confirmatory factor analysis (CFA) results indicated that the four-factor model demonstrated good fit (χ^2^/df = 3.688, RMSEA = 0.076, NFI = 0.959, CFI = 0.970, GFI = 0.904), meeting acceptance criteria and confirming the validity of the four-factor theoretical structure and dimensionality of the scales. Secondly, educational LLM agents (CR = 0.943, AVE = 0.805), school support (CR = 0.926, AVE = 0.757), teacher self-efficacy (CR = 0.955, AVE = 0.809), and teachers’ curriculum content creation (CR = 0.957, AVE = 0.847) all exhibited composite reliability (CR) ≥ 0.7 and average variance extracted (AVE) ≥ 0.5. This indicated strong item aggregation under the same construct, effectively capturing core conceptual content. Finally, calculations confirmed that all constructs satisfied the criterion ‘AVE root square > correlation coefficients with other constructs,’ thereby meeting the Fornell-Larcker criteria. The HTMT values were: 0.638 for educational LLM agents and school support; 0.731 for teacher self-efficacy; 0.71 for teachers’ curriculum content creation; school support exhibited HTMTs of 0.698 and 0.619 with teacher self-efficacy and teachers’ curriculum content creation, respectively; teacher self-efficacy showed an HTMT of 0.694 with teachers’ curriculum content creation. Their Heterogeneity-to-Homogeneity Ratios (HTMTs) were all <0.85, confirming clear construct boundaries and the absence of dimensional overlap.

#### 4.2.2. Measurement Model

We conducted confirmatory factor analysis (CFA) to evaluate the discriminant validity of the variables. We specified the baseline model (M1) as a four-factor model, comprising Educational LLM Agent Use, School Support, Teacher Self-Efficacy, and Teachers’ Curriculum Content Creation. On this basis, we have constructed five alternative models:

Model 1: A three-factor model that combines Educational LLM Agent Use and School Support into one factor, with Teacher Self-Efficacy and Teachers’ Curriculum Content Creation as separate factors.

Model 2: A three-factor model that combines Educational LLM Agent Use and Teacher Self-Efficacy into one factor, with School Support and Teachers’ Curriculum Content Creation as separate factors.

Model 3: A three-factor model that combines School Support and Teacher Self-Efficacy into one factor, with Educational LLM Agent Use and Teachers’ Curriculum Content Creation as separate factors.

Model 4: A two-factor model that combines Educational LLM Agent Use, Teacher Self-Efficacy, and School Support into one factor, with Teachers’ Curriculum Content Creation as a separate factor.

Model 5: A one-factor model that merges all variables into a single factor.

Model comparison results showed that the one-factor model (χ^2^/df = 10.817, CFI = 0.884, TLI = 0.867, GFI = 0.692, RMSEA = 0.146, SRMR = 0.0466) performed worst across all indices—indicating that a single-factor structure did not fit the data. By contrast, the baseline four-factor model exhibited satisfactory fit indices (χ^2^/df = 3.688, RMSEA = 0.076, NFI = 0.959, CFI = 0.970, GFI = 0.904) and outperformed all competing models. This demonstrates that the four constructs—Educational LLM Agent Use, School Support, Teacher Self-Efficacy, and Teachers’ Curriculum Content Creation—have good structural validity and can be meaningfully distinguished as independent factors (details are shown in Table 2).

### 4.3. Data Analysis

We conducted quantitative analyses using SPSS 26.0. First, we used descriptive statistics and correlation analyses to overview sample characteristics. Secondly, we employed SPSS AMOS 26.0 and the Maximum Likelihood (ML) estimator to conduct structural equation modeling (SEM) analysis in order to test our proposed hypotheses. Finally, we examined the mechanisms underlying the relationship between Educational LLM Agent Use and Teachers’ Curriculum Content Creation, with a focus on the mediating roles of School Support and Teacher Self-Efficacy.

## 5. Results

### 5.1. Common Method Bias Test

Because this study used multiple scales completed by the same participants, we examined the potential for common method bias. Following Zhou and Long’s (2004) recommendations, the methods of “Harman single factor test” and “controlling unmeasured single method potential factor” were used to test the common method bias. When all items were loaded onto a single common factor, the fit indices were poor: χ^2^/df = 10.817, CFI = 0.884, TLI = 0.867, GFI = 0.692, RMSEA = 0.146, and RMR = 0.038. In contrast, the four-factor model produced substantially better fit indices (χ^2^/df = 3.688, RMSEA = 0.076, NFI = 0.959, CFI = 0.970, GFI = 0.904, RMR = 0.018). Subsequently, after adding the method factor to the four-factor model, the model’s fit indices were as follows:χ^2^/df = 3.077, CFI = 0.977, TLI = 0.972, GFI = 0.921, RMSEA = 0.067, and RMR = 0.063. The results show that ΔCFI = 0.007, ΔTLI = 0.008 < 0.1, and ΔRMSEA = 0.009, ΔRMR = 0.045 < 0.05, indicating that there is no significant common method bias in the measurements.

### 5.2. Descriptive Statistics and Correlation Analysis

The mean scores of the study variables ranged from 2.98 (for School Support) to 3.20 (for Teachers’ Curriculum Content Creation)—values close to the scale’s midpoint of 3. This indicated that teachers generally held moderately high perceptions of School Support, Educational LLM Agent Use, Teacher Self-Efficacy, and Curriculum Content Creation. The standard deviations were all around 0.8, reflecting moderate data dispersion and a relatively even distribution.

Correlation analysis further revealed significant positive associations between all variables (*p* < 0.01), with strong correlations observed among Educational LLM Agent Use, Teacher Self-Efficacy, School Support, and Teachers’ Curriculum Content Creation. These results confirmed that the variables are closely related, providing an empirical basis for further analyzing how Educational LLM Agent Use influences curriculum content creation (Table 3).

However, the high correlation coefficients among variables readily give rise to multicollinearity issues. Consequently, this study employed educational LLM agents, school support, and teacher self-efficacy as independent variables, with teachers’ curriculum content creation as the dependent variable, for further validation. Results indicated that the variance inflation factors (VIFs) for educational LLM agents (VIF = 5.626), school support (VIF = 3.205), and teacher self-efficacy (VIF = 7.04) were all <10. This suggested that despite high inter-variable correlations, the current regression model exhibits no severe multicollinearity interference, and the estimated effects of each variable on the dependent variable possess a degree of reliability.

### 5.3. Testing the Chain Mediation Model of School Support and Teacher Self-Efficacy

#### 5.3.1. Regression Analyses

Table 4 presents the regression results for the relationships among the variables. In the regression model predicting School Support, Educational LLM Agent Use had a standardized coefficient of 0.777 (*p* < 0.001), indicating a significant positive predictive effect. In the regression model predicting Teacher Self-Efficacy, both School Support (β = 0.311, *p* < 0.001) and Educational LLM Agent Use (β = 0.664, *p* < 0.001) exerted significant positive effects. Educational LLM Agent Use showed a stronger influence. In the regression model predicting Teachers’ Curriculum Content Creation, the effect of School Support (β = 0.63, *p* > 0.05) was not significant. Both Educational LLM Agent Use (β = 0.690, *p* < 0.001) and Teacher Self-Efficacy (β = 0.181, *p* < 0.01), however, significantly and positively predicted curriculum content creation (Table 4).

#### 5.3.2. Mediation Analyses

In the chained mediation model analysis conducted within this study, standardized data was employed to ensure the accuracy and robustness of mediation effect testing. The PROCESS v4.1 macro was utilized to perform the relevant tests, with the following parameter settings: A 95% confidence level was selected, employing BCa (bias-corrected accelerated) confidence intervals to better accommodate potential non-normal data distributions; Simultaneously, a random seed of 20241211 was specified to guarantee the replicability of the testing process, thereby providing reliable statistical grounds for determining the significance of chained mediating effects. In addition, we examined the mediating effects of School Support and Teacher Self-Efficacy using the bootstrap method. Moreover, this study constructed a targeted semi-structured interview outline to further explore the intrinsic relationships among various variables.

**Figure 2:**
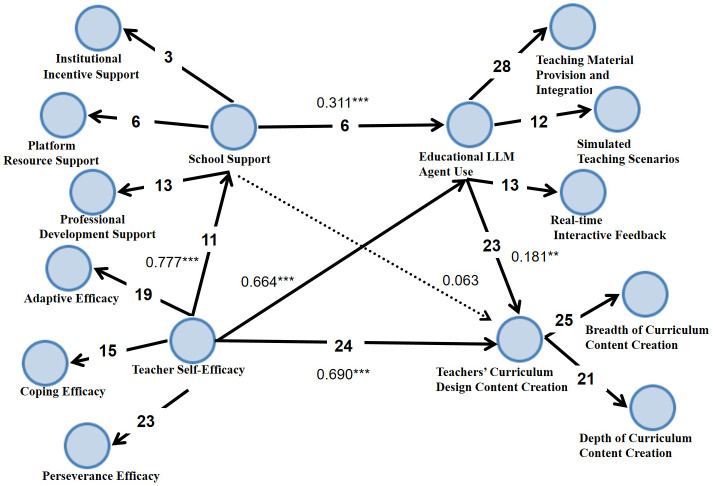
Chain Mediation Effect Model and Interview Frequency. ** *p* < 0.01, *** *p* < 0.001.

In the qualitative analysis section, this study employed content analysis, a semi-quantitative research method that enables repeatable, valid inferences from texts (or other meaning-bearing entities such as videos) to their contextual usage. It objectively, systematically, and quantitatively describes explicit communication content. Utilizing this approach, the study conducted objective, systematic, and quantitative content coding and analysis of materials related to teachers’ use of educational LLM agents in curriculum content creation. The specific process strictly adhered to the six stages of content analysis methodology: establishing research objectives, defining the research population and selecting units of analysis, designing the analytical dimension system, sampling and quantifying the materials, and conducting evaluative recording and analytical inference. Throughout, standardized procedures inherent to content analysis were applied, including dual coding, consistency testing and reporting, and resolving discrepancies through discussion. In the sample selection phase, the research team first selected 37 teachers who left their WeChat IDs in the last optional question from 464 valid questionnaires. Following up and communicating with them, 23 teachers ultimately volunteered to participate in this semi-structured interview, providing ample first-hand data for qualitative analysis (Figure 2).

Total effect: Educational LLM Agent Use had a total effect of 0.902 on Teachers’ Curriculum Content Creation (95% CI [0.8569, 0.9353]), confirming a substantial overall influence.

Direct effect: The direct effect was 0.6895 (95% CI [0.5983, 0.7807]), accounting for 76.4% of the total effect. This indicates that Educational LLM Agent Use directly predicts curriculum content creation, providing strong support for Hypothesis H1. As Respondent 19 stated: Our school secured a pilot program for an educational large model. Through such models, we efficiently obtained substantial course design content creation materials, enriching our teaching content diversity. Teachers leveraging educational LLM agents to enhance curriculum content creation fully validates Hypothesis 1.

Indirect effect via School Support: The indirect path “Educational LLM Agent Use → School Support → Curriculum Content Creation” was not significant (95% CI [−0.0258, 0.1357]), as the confidence interval included zero. Hypothesis H2 was therefore not supported. In qualitative research, Respondent 7 reflected: “When using AI agents to assist in designing course teaching content, the Spark Teacher Assistant AI agent recommended during school training proved unhelpful. I was designing a junior secondary school Rainbow Fountain experiment plan at the time, and the content provided by the Spark Teacher Assistant recommended by the school was not what I needed. It was less useful than the “Physics, Chemistry and Biology Experiment Inquiry Design” personalized AI platform recommended to me by my apprentice. This platform could provide the experiment’s purpose, a list of experimental materials, experimental steps, expected results, experimental conclusions, and safety precautions. Notably, the materials list was presented in tabular format, categorized into containers, reagents, tools, and auxiliary materials, with specific items and safety warnings included. This demonstrates that resources provided by schools, if they fail to precisely match teachers’ needs, cannot effectively drive the creation of curriculum content. This further explains why Hypothesis 2 was not significant.

Indirect effect via Teacher Self-Efficacy: The indirect path “Educational LLM Agent Use → Teacher Self-Efficacy → Curriculum Content Creation” was significant, with an effect size of 0.1200 (95% CI [0.0364, 0.2113])—accounting for 13.30% of the total effect. This means Educational LLM Agent Use enhances Teacher Self-Efficacy, which in turn facilitates curriculum content creation. As Respondent 14 noted: “I used the teaching materials provided by Kimi and find their quality increasingly high, with reference sources also provided. The optimization following follow-up inquiries has also improved significantly. I frequently shared exemplary application cases within our teaching research group chat, and later the year group even invited me to share my experience, which gave me a real sense of pride. Moreover, during the teaching research experience sharing session, there was deeper case discussion centered around the content design of my elective course, which actually proved very helpful for further developing that elective.” Teachers’ use of educational LLM agents enhances their sense of self-efficacy, indirectly driving curriculum content creation, thereby fully validating Hypothesis 3.

Chain mediation effect: The path “Educational LLM Agent Use → School Support → Teacher Self-Efficacy → Curriculum Content Creation” produced a significant chain mediation effect (effect size = 0.0437; 95% CI [0.0146, 0.0763]), accounting for 4.8% of the total effect. While smaller in magnitude, this pathway shows School Support indirectly contributes to curriculum content creation by strengthening Teacher Self-Efficacy. As interviewee 19 noted, ‘The school secured a pilot for a large-scale educational model,’ and interviewee 14 remarked, ‘Using Kimi yielded valuable application cases, which we later shared as best practices—a source of considerable pride.’ The use of educational LLM agents drives institutional support for enhancing teacher self-efficacy, ultimately elevating curriculum content creation capabilities. This fully validates Hypothesis 4.

The total indirect effect was 0.2125 (95% CI [0.1036, 0.3338]), accounting for 23.6% of the total effect. These results confirm that Educational LLM Agent Use not only directly shapes teachers’ curriculum content creation but also exerts indirect effects through School Support and Teacher Self-Efficacy (Table 5).

Overall, the findings from the chain mediation model (Figure 2) strongly confirmed that Educational LLM agent use significantly predicts Teachers’ Curriculum Content Creation—both directly (β = 0.690, *p* < 0.001) and indirectly through mediating pathways. Specifically, Educational LLM Agent Use positively predicted both School Support (β = 0.777, *p* < 0.001) and Teacher Self-Efficacy (β = 0.664, *p* < 0.001). Moreover, School Support exerted a significant positive effect on Teacher Self-Efficacy (β = 0.311, *p* < 0.001), which means supportive school resources boost teachers’ confidence in their teaching practices.

Through quantitative analysis, while School Support did not have a significant direct effect on curriculum content creation (β = 0.063, *p* > 0.05), the overall mediation mechanism remains clear: Educational LLM agent use first fosters School Support, which in turn strengthens Teacher Self-Efficacy, ultimately promoting teachers’ curriculum content creation indirectly. Meanwhile, qualitative analysis revealed that the multi-scenario applications of educational LLM agents, specifically material provision and integration (mentioned 23 times), simulated teaching scenarios (11 mentions), and real-time interactive feedback (19 mentions), directly enhance teachers’ curriculum content creation capabilities across both breadth (12 mentions) and depth (16 mentions) dimensions. In addition, the application of educational LLM agents indirectly supports curriculum content creation by fostering three core sub-themes of teacher self-efficacy: adaptive efficacy (mentioned 11 times), coping efficacy (mentioned 9 times), and perseverance efficacy (mentioned 15 times). Additionally, educational LLM agent applications bolster teachers’ self-efficacy by promoting institutional incentives (mentioned 7 times), platform resource support (mentioned 12 times), and teaching research training (mentioned 17 times), thereby indirectly laying the groundwork for curriculum content creation. Therefore, the frequency statistics and thematic analysis results obtained from the qualitative research phase not only form a good correspondence with the quantitative research conclusions but also supplement specific practical contexts for this mediating mechanism, thereby further consolidating the credibility of this research finding.

In summary, Educational LLM Agent Use influences Teachers’ Curriculum Content Creation through both direct and indirect pathways. These results underscore the importance of strengthening School Support and enhancing Teacher Self-Efficacy to maximize the role of educational LLM agents in fostering innovative teaching practices.

## 6. Discussion

This study systematically examined the pathways and underlying mechanisms by which educational LLM agent use influences teachers’ curriculum content creation. The findings revealed that educational LLM agent use not only exerts a direct impact on teachers’ content creation but also enhances it indirectly—first through teacher self-efficacy alone, and second through the sequential mediation of school support and teacher self-efficacy. These results advanced our understanding of how educational LLM agent use shapes teachers’ professional practices and lay a theoretical foundation for optimizing teachers’ curriculum content creation.

### 6.1. The Relationship Between Educational LLM Agent Use and Teachers’ Curriculum Content Creation

The results supported Hypothesis H1: Educational LLM agent use significantly and positively predict teachers’ curriculum content creation (total effect = 0.8961, *p* < 0.001), with the direct effect accounting for 76.44% of the total effect. This indicates that nearly 80% of the promotional effect of educational LLM agents on teachers’ course content innovation is realized directly using technology itself. While the indirect effect is statistically significant, it accounts for only 23.56% of the total effect, a relatively small proportion that stands out in comparison to the direct effect. This disparity in effect size holds significant implications: it provides crucial evidence for identifying the core pathway through which educational LLM agents influence teachers’ course content innovation. Compared to indirect effects mediated by intermediary variables, the direct application of educational LLM agents plays a more pivotal role in driving teachers’ course content innovation. This finding aligned closely with Song et al.’s (2025) research, which emphasized that interactive agent tools effectively enhance teachers’ instructional practices. It thus confirmed the core value of intelligent technologies as “enablers” in teacher professional development. For instance, many teachers have adopted innovative approaches to integrating AI tools like ChatGPT into their practice (Oster et al., 2024). With AI support, teaching can be designed more effectively to deliver engaging lessons and develop personalized materials, helping students achieve academic success (Meron & Araci, 2023).In these practical scenarios, the direct application of educational LLM agents consistently serves as the pivotal element in enhancing teachers’ capacity for curriculum innovation, further corroborating the research findings that ‘direct effects predominate’.

Furthermore, educational LLM agents influence teachers’ curriculum content creation across multiple dimensions in substantial ways. First, in breadth, generative AI integrates interdisciplinary knowledge and recommends diverse instructional resources (e.g., external knowledge bases and digital tools), enabling teachers to expand curriculum content coverage (Mu et al., 2025). For example, Interviewee 19 noted that their school gained access to a pilot program for a large educational model, which provided dedicated accounts and passwords. With this tool, teachers efficiently obtained a wide range of curriculum content creation materials, thereby diversifying teaching content, showing how agents support content breadth. Second, in great detail, agents can analyze learning data, deconstruct complex concepts, and guide teachers to enhance the logical coherence and relevance of instructional content. For instance, retrieval-augmented generation (RAG) techniques grant access to cutting-edge disciplinary content or in-depth explanations (Yao & Wang, 2024). Interviewee 14 shared that DeepSeek provided high-quality instructional resources with cited sources, and its output grew more refined with optimization. This not only enhanced elective course development but also stimulated further professional dialogue during teaching research meetings, reflecting how agents contribute to content depth.

The link between educational LLM agent uses and curriculum content creation also manifests in process optimization. Agents can assist teachers with tasks like content storage, memory retrieval, and dynamic adjustment (Ding et al., 2025)—for example, automatically generating learning objectives or optimizing teaching activity design. These capabilities let teachers focus on innovation and appropriateness in curriculum content. Moreover, by simulating teaching scenarios and providing real-time feedback, agents help teachers balance disciplinary rigor with student needs during content creation (Theeuwes et al., 2025), ultimately improving curriculum design quality and efficiency. Interviewee 6 further noted that teachers actively shared application cases in research groups, where in-depth discussions on elective course content design took place during experience-sharing activities. These practices clearly demonstrate how educational LLM agents enhance the depth of curriculum content creation. This not only enriches curriculum content but also aligns with Norton et al.’s (2022) perspective on interdisciplinary collaboration, suggesting that by expanding resource channels and fostering teacher collaboration, educational LLM agents can further deepen curriculum design substance and broaden its scope.

### 6.2. The Influence of School Support and Teacher Self-Efficacy

The results indicated that Hypothesis H2 is not supported, which suggests the independent mediating effect of school support between educational LLM agent use and teachers’ curriculum content creation is not significant. This lack of significance may stem from multiple external factors and potential limitations at the theoretical model level, necessitating in-depth analysis from both internal and external dimensions. From the perspective of external support for practice, school support for educational LLM agents often remains at the infrastructure level—for instance, procuring tools or organizing basic operational training—without extending to the critical stages of deep integration with curriculum design (Hazzan-Bishara et al., 2025). By contrast, the MECC project at the University of Michigan, studied by Norton et al. (2022), received substantial university funding to hire professional project managers and cover student travel expenses. This funding ensured the project could be implemented in depth. These comparisons suggest that if schools strengthened support through financial investment and professional staffing, the mediating role of school support between educational LLM agent use and curriculum content creation might become more pronounced. Concurrently, this non-significant finding prompts self-critical reflection, necessitating acknowledgement of potential inherent flaws within the theoretical model and hypotheses themselves. Hypothesis H2 was constructed based on the causal logic of ‘educational LLM agents utilization → school support → teacher curriculum content creation,’ yet failed to adequately account for the complexity of causal relationships among these three elements, reverse causality effects may exist, such as higher-level demands for teacher-created curriculum content driving enhanced school support, or critical variables that could moderate these relationships, such as teachers’ technological acceptance or subject-specific teaching characteristics, were omitted. This resulted in the model’s inadequate fit to real educational contexts. Such limitations in theoretical construction may have further diminished the manifestation of the mediating effect of school support. Consequently, this non-significant finding not only reflects the superficial nature of current school support practices but also exposes limitations in the theoretical model’s causal framework and variable dimensions. This provides crucial insights for subsequent research in model refinement and hypothesis adjustment.

By contrast, teacher self-efficacy exerts a significant mediating effect between educational LLM agent use and teachers’ curriculum content creation, which supports Hypothesis H3. The application of intelligent technologies can significantly strengthen teachers’ self-efficacy, and this enhanced self-efficacy in turn improves their ability to develop curriculum content (Song et al., 2025). From the lens of social cognitive theory (Bandura, 1977), individuals’ beliefs about their own capabilities profoundly shape their behaviors, emotions, and cognitions. Huang et al. (2020) also found that teacher self-efficacy has a significant positive impact on teacher professional learning (TPL), and this impact further promotes innovative practices.

Self-efficacy, defined as a belief in one’s own capabilities (Cai & Tang, 2021), takes shape in the context of agent use as teachers’ confidence in applying technologies to content creation. Teachers with higher self-efficacy are more likely to actively explore agent functions (e.g., leveraging multimodal resources, optimizing instructional assessment), and this exploration enriches both the breadth and depth of curriculum content (Ding et al., 2025). For example, confident teachers may use agents to generate personalized assignments or interdisciplinary cases, which enhances the relevance of curriculum content. By contrast, school support, such as training resources and technological infrastructure, has a weaker direct influence on curriculum content creation. This may stem from the fact that school support exerts its influence more effectively through indirect pathways, rather than directly shaping content creation itself (Cai & Tang, 2021). Nevertheless, qualitative interviews also underscore the value of school support. Interviewee 19, for instance, mentioned that their school gained access to a pilot project for large educational models; this project created opportunities for teachers to engage with and use agents. Even so, such influence manifests primarily through mediating mechanisms, not direct effects.

### 6.3. The Chain Mediation of School Support and Teacher Self-Efficacy

The results supported Hypothesis H4. Although school support has no significant independent mediating effect, the chain mediation pathway “educational LLM agent use → school support → teacher self-efficacy → teachers’ curriculum content creation” is significant. This finding suggested that school support does not influence curriculum design in isolation; instead, it works in synergy with teacher self-efficacy. When teachers are in an environment characterized by institutional trust, collegial support, and leadership encouragement, their self-efficacy improves. This enhanced self-efficacy then motivates them to participate more actively in various teaching activities (Keedy et al., 2001). Moreover, research has shown that distributed leadership and teacher collaboration significantly boost both teacher self-efficacy and professional well-being (Wang et al., 2025). Additionally, school climate factors, including institutional integrity, principal influence, care and compassion, support systems, school culture, and academic emphasis, all shape teachers’ self-efficacy (Fang & Qi, 2023).

Existing literature further showed a broad consensus that school support must integrate both technical and psychological dimensions. Specifically, school support, including organizing AI-focused teaching seminars and providing technical assistance, creates favorable conditions for teachers to master agent-based tools. It also strengthened their confidence in using technology, which is their self-efficacy (Ding et al., 2025). Interview data also highlighted that some schools gained access to pilot programs for large educational models, which provided teachers with dedicated accounts and passwords. These measures allowed teachers to use and adapt to educational LLM agents earlier and to efficiently acquire diverse resources for curriculum content creation, ultimately enriching their teaching materials. This case clearly illustrates how school support contributes positively to the breadth of curriculum content creation by enhancing teacher self-efficacy. The chain mediation effect of school support thus acts as a critical link between educational LLM agent use and curriculum design, offering clear direction for schools to optimize their support strategies.

Accordingly, schools should prioritize building a comprehensive, multi-level support system. This system should not only provide material guarantees but also offer encouragement and recognition at the psychological level. This dual approach fosters teachers’ intrinsic motivation and strengthens their self-efficacy. Higher self-efficacy, in turn, promotes the active use of agents for content creation. Examples include expanding curriculum breadth by integrating interdisciplinary resources and deepening content through the precise deconstruction of knowledge points (Mu et al., 2025). The core logic of chain mediation lies in transforming school support—an “external resource”—into actual behavior (i.e., content creation) through teachers’ “internal beliefs” (i.e., self-efficacy). For instance, when schools’ agent-use training successfully enhances teachers’ technological confidence, teachers are more likely to proactively use agents to generate innovative teaching content (Cai & Tang, 2021; Yao & Wang, 2024). In this way, the pathway “school support → teacher self-efficacy → curriculum content creation” effectively translates support into improved content creation quality.

## 7. Limitations and Future Directions

Despite the strong application potential of educational LLM agents, their current development still faces certain limitations.

Firstly, Han (2024) pointed out that issues like data privacy and algorithmic bias remain significant challenges, and these challenges constrain the deeper integration of educational LLM agents into education.

Secondly, this study did not incorporate certain control variables (such as demographic variables), potentially introducing minor omission bias. However, adding supplementary control variables increases model complexity, and pre-testing indicates these variables exert no significant influence on core pathways. Consequently, establishing the baseline model remains a priority. Future research may employ multi-group SEM to validate the cross-sample robustness of the baseline model or construct cross-lagged models using longitudinal data to further enhance the reliability of causal inference.

Thirdly, this study exhibited certain limitations in terms of sample size and disciplinary coverage, which to some extent constrain the generalizability of its conclusions. Firstly, the geographical distribution of the sample is markedly imbalanced, with research data predominantly sourced from economically developed coastal regions. The proportion of survey samples from Western and less developed areas is extremely low, making it difficult to fully reflect the operational mechanisms of research variables across diverse educational settings. It also fails to comprehensively reveal the potential impact of regional developmental disparities on the core research questions. Secondly, the selection of subject areas is singularly focused. Current research samples and data concentrate exclusively on science disciplines, excluding subjects such as the humanities and languages. Given the significant differences in teaching methodologies, learning contexts, and teacher–student interaction dynamics across disciplines, the findings struggle to be transferred to the broader educational landscape encompassing all subject areas. Therefore, future research may be optimized and expanded in two respects. Firstly, efforts should be made to broaden the geographical coverage of the sample, with particular emphasis on supplementing survey data from western and less developed regions. By comparing samples across multiple regions and developmental levels, the cross-regional applicability of this study’s conclusions may be validated, whilst simultaneously exploring the interactive effects between regional development factors and core research variables. Secondly, disciplinary boundaries should be transcended by incorporating the humanities and language disciplines into the research scope. Comparing the adaptability of research models across different academic fields will enhance the theoretical framework’s disciplinary universality, thereby providing more comprehensive empirical support for constructing an education intervention system with full disciplinary coverage.

Fourthly, the scales employed in this study exhibit high Cronbach’s alpha values, potentially indicating redundancy. However, owing to constraints on sample accessibility and the unique timing of the research, it may not be feasible to revise the scales. Consequently, future research will incorporate semantic differentiation screening and reverse-scored items during the scale design phase. This approach aims to broaden the expressive dimensions of items while preserving the core construction’s essence, thereby balancing internal consistency with the richness of construct representation.

Last but not least, other factors, such as those related to schools and local education authorities, may influence how strongly teachers’ curriculum content creation is affected. Therefore, future research should further investigate the independent roles and interactive effects of factors at the educational administration and school levels. Such efforts will help uncover the underlying mechanisms connecting educational LLM agent use and teachers’ curriculum content creation from a wider perspective.

At the same time, educational LLM agents keep significant promises. Recent studies have shown that teaching agents, which act as virtual assistants, can support teachers’ pedagogical practices; future research may further enhance their role in improving instruction (Khlaif et al., 2024). Additionally, Küchemann et al. (2025) explored the integration of AI agents with large language models in education, highlighting that this integration has the potential to usher in an era of personalized AI—one that enables teachers to explore new instructional approaches. Looking ahead, educational LLM agents are expected to evolve to be more personalized, intelligent, and human-centered, integrating deeply with multiple technologies to drive transformative breakthroughs in education. These advancements will ultimately contribute to achieving more equitable, efficient, and high-quality education.

## 8. Conclusions

As education’s digital transformation deepens, educational LLM agents have become increasingly integrated into teachers’ curriculum content creation practices. Teachers, however, face multiple challenges: difficulty adapting to new tools, incomplete school-level support systems, and fluctuations in self-efficacy. If unresolved, these issues may gradually develop into critical barriers, ones that hinder the effectiveness of instructional innovation and the advancement of teachers’ professional development. Nevertheless, this study underscored the transformative value of educational LLM agents in addressing such barriers. Through the chain mediating effect of school support and teacher self-efficacy, educational LLM agents not only reshape curriculum design logic and empower content creation innovation but also foster collaborative vitality within educational contexts. Moreover, Bandura’s social cognitive theory initially focused on interpersonal interaction scenarios, with its core triadic interaction determinism (individual factors, behavior, environment) long employed to explain cognitive and behavioral linkages among human groups (Bandura, 1986). In recent years, with the proliferation of artificial intelligence technologies, some scholars have extended the theory’s application to human–computer interaction domains (Guan et al., 2025). However, existing research has scarcely addressed mutual cognitive facilitation between humans and personalized educational LLM agents. Consequently, this study transcends social cognitive theory’s traditional interpersonal interaction framework while expanding the boundaries of human–computer interaction research. By incorporating ‘cognitive interaction between humans and personalized educational LLM agents’ into theoretical analysis, it provides novel empirical support for the theoretical evolution of social cognitive theory in the era of intelligent education.

This study’s findings showed that educational LLM agent use has a significant positive effect on teachers’ curriculum content creation. As digital professional resources, educational agents reduce teachers’ workload, letting them shift focus away from routine administrative tasks and concentrate on innovation (Kohnke & Ulla, 2024). Regarding teacher self-efficacy’s mediating role, the results indicate that it makes a significant positive contribution to linking educational LLM agent use with curriculum design innovation. When teachers recognize their ability to use agents to overcome curriculum design inertia and achieve instructional innovation, they are more willing to adopt technological tools and integrate them deeply into pedagogical practice (Zhu et al., 2025). More importantly, while school support had a limited independent mediating effect, the chain mediation of “school support → teacher self-efficacy” proved significant. This result underscores that educational LLM agents’ ability to empower curriculum design innovation depends on the dual logic of external support and internal belief, and only then can technology’s full value be realized.

In summary, educational LLM agents have opened new dimensions for teachers’ curriculum content creation. Sustained educational innovation can only be achieved by continuously optimizing school support systems, enhancing teachers’ self-efficacy, and ensuring technology serves as a true enabler rather than a new burden. In optimizing the school support system, tangible assistance should be provided based on teachers’ practical requirements when utilizing educational LLM agents. For instance, to address issues such as insufficient account permissions and limited operational skills encountered by teachers when using educational LLM agents, schools may provide dedicated licensed accounts to ensure full access to core functionalities like curriculum resource analysis and student learning data tracking. For individualized technical challenges, peer-led workshops could be organized, inviting teachers proficient in educational LLM agent applications to share practical experience. To enhance teachers’ self-efficacy, targeted guidance should be grounded in qualitative data. By analyzing classroom observation records, teacher interview feedback, and student learning journals, one can identify strengths and weaknesses in teachers’ educational LLM agent application. For those adept at using educational LLM agents for learning analytics but struggling with resource integration, recommend built-in resource matching templates alongside specialized training on resource selection and restructuring techniques. For teachers proficient in technical operations but lacking experience in translating data insights into teaching strategies, case-based guidance can be provided, demonstrating how other educators design differentiated teaching activities based on the cognitive weaknesses identified by educational LLM agents. In turn, this will allow educational LLM agents to deeply empower teacher development, ultimately yielding teaching outcomes with greater contemporary relevance and laying a solid foundation for building a future-oriented educational ecosystem.

## Figures and Tables

**Table 1:** Demographic characteristics of surveyed lower secondary STEM teachers (*n* = 464).

Variable	Category	Frequency	Percentage
Gender	Male	149	32.1%
	Female	315	67.9%
Household Registration	Rural	350	75.4%
	Urban	114	24.6%
Teaching Grade	Grade 7	215	46.4%
	Grade 8	147	31.7%
	Grade 9	102	22.0%
Teaching Subject	Science	188	40.5%
	Information Technology	113	24.4%
	Mathematics	163	35.1%

**Table 2:** Summary of Model Fit Indices and Model Comparisons (*n* = 464).

Model	χ^2^	df	χ^2^/df	CFI	TLI	NFI	GFI	RMSEA	SRMR	Δχ^2^
Baseline Model (M1)	416.794	113	3.688	0.970	0.964	0.959	0.904	0.076	0.0234	
Model 1	868.401	116	7.486	0.925	0.912	0.915	0.783	0.118	0.0409	359.606
Model 2	570.135	116	4.915	0.955	0.947	0.944	0.866	0.092	0.0288	153.341
Model 3	763.599	116	6.582	0.936	0.925	0.925	0.807	0.110	0.0349	346.805
Model 4	961.679	118	8.150	0.916	0.903	0.906	0.768	0.124	0.0418	544.885
Model 5	1287.207	119	10.817	0.884	0.867	0.874	0.692	0.146	0.0466	870.413

**Table 3:** Descriptive statistics and correlations among variables (*n* = 464).

Variable	M ± SD	1. Educational LLM Agent Use	2. School Support	3. Teacher Self-Efficacy
1. Educational LLM Agent Use	3.17 ± 0.814			
2. School Support	2.98 ± 0.825	0.777 **		
3. Teacher Self-Efficacy	3.11 ± 0.809	0.905 **	0.827 **	
4. Teachers’ Curriculum Content Creation	3.20 ± 0.808	0.902 **	0.748 **	0.857 **

Note: ** *p* < 0.01. All correlations are reported for this study.

**Table 4:** Regression analysis of relationships among variables in the chain mediation model.

Regression Path		Overall Fit Indices			Predictors (IVS)	
Outcome Variable (DV)	Predictor Variable (IV)	*R*	*R* ^2^	*F*	*β*	*t*
School support	Educational LLM Agent Use	0.777	0.604	704.445	0.777	26.541 ***
Teacher Self-efficacy	Educational LLM Agent Use	0.926	0.858	1392.108	0.664	23.796 ***
	School support				0.311	11.146 ***
Teachers’ Curriculum Content Creation	Educational LLM Agent Use	0.908	0.824	717.136	0.690	14.856 ***
	School support				0.063	1.793
	Teacher Self-efficacy				0.181	3.481 **

Note: ** *p* < 0.01, *** *p* < 0.001. Apply to all statistical results reported in this study.

**Table 5:** Mediation effect analysis.

Pathway	Indirect Effect	Bootstrap SE	95% CI	Significance	Proportion of Effect
Total Effect	0.902	0.0200	0.8569~0.9353	Significant	100%
Direct Effect	0.6895	0.0464	0.5983~0.7807	Significant	76.4%
Indirect Effect	0.2125	0.0592	0.1036~0.3338	Significant	23.6%
Educational LLM Agent Use → School Support → Teachers’ Curriculum Content Creation	0.0488	0.0412	−0.0258~0.1357	Not Significant	\
Educational LLM Agent Use → Teacher Self-Efficacy → Teachers’ Curriculum Content Creation	0.1200	0.0446	0.0364~0.2113	Significant	13.3%
Educational LLM Agent Use → School Support → Teacher Self-Efficacy → Teachers’ Curriculum Content Creation	0.0437	0.0159	0.0146~0.0763	Significant	4.8%

## Data Availability

The data presented in this study are only available on request from the corresponding author due to confidentiality restrictions.

## Appendix A. Survey Instruments in the Study

Questionnaire survey of educational LLM agents applied to teachers’ professional development.

Dear teacher:

Hello! In order to gain a deeper understanding of your cognition, usage status and related attitudes towards agent tools, and explore the application value and potential of agents in education and teaching scenarios, we conducted this questionnaire survey. This survey is anonymous, and all data is only used for academic research and is strictly confidential. It is estimated that it will take you 5–7 min to fill out the questionnaire, please answer truthfully according to your actual situation

Thank you for your support and cooperation in your busy schedule!

**1. Basic information**
  Your gender is: □ Male □ Female
  Your domicile:
  □ Rural □ townships □ counties □ prefecture-level cities □ provincial capitals □ municipalities directly under the Central Government
  The middle school grades you teach is:
  □ 1st □ 2nd □ 3rd grade □ Cross-grade teaching (please specify: ______)
  The subjects you teach is:
  □ Chinese □ Mathematics □ English □ Physics □ Chemistry □ Biology □ History
  □ Geography □ Politics (Ethics & Rule of Law) □ Music □ Fine Arts □ Physical Education
  □ Information Technology □ Others (please specify: ______)
  **2. Agent tools and functional cognition**
  Please hit “√” after the option that matches your situation, you can select multiple options:
  I understand the tool platform of the agent: □ Domestic: Baidu Wenxin Agent
  □ Domestic: iFLYTEK Spark Platform □ Domestic: Tencent Hunyuan Platform □ Others (please specify: ______)
  I understand the functions of the following agents: □ Text-to-Video
  Function □ Text-to-Document □ Answering Function □ Text-to-Image
  Diagram Function □ Audio Clip Function □ Code Generation Function □ Others (Please Specify: ______)
  **3. Attitude towards the use and operation of agent tools**
  The meaning of each alternative answer is as follows (same below):
  5 Very true: means that this statement holds true for you in almost all cases;
  4 Conformity: means that under normal circumstances, this statement is consistent with you;
  3 Uncertainty: means that in half of the cases, this statement is consistent with you;
  2 Non-conformity: means that under normal circumstances, this statement is not in accordance with you;
  1 Very inconsistent: means that in almost all cases this statement is inconsistent with you

**Scale Problem**	**Degree of Conformity**				
1. I will use an educational LLM agent for personalized learning.	1	2	3	4	5
2. Educational LLM agents will enhance my teaching practice and interaction with digital resources.	1	2	3	4	5
3. When using an educational LLM agent, I feel that technology can help me alleviate some of the teaching tasks that I don’t think are ideal.	1	2	3	4	5
4. The Educational LLM Agent Application takes on important ancillary tasks that I cannot complete due to time constraints.	1	2	3	4	5
5. I will be hiring an educational LLM agent because it has a significantly higher adoption rate among teaching staff and peers at my institution.	1	2	3	4	5
6. Both institutions and faculty teams support the use of LLM agents in education.	1	2	3	4	5
7. My master teacher will provide strong support in my use of educational LLM agents in course design content creation.	1	2	3	4	5
8. Overall, the agency facilitates support for the implementation of educational LLM agents.	1	2	3	4	5
9. I am confident in executing curriculum design content creation efficiently.	1	2	3	4	5
10. I am confident in the process of creating course design content using an educational LLM agent.	1	2	3	4	5
11. If you want to use an education-grade language model agent to produce course design content, I believe you can succeed.	1	2	3	4	5
12. I am confident in using an educational LLM agent to improve the quality of course design content.	1	2	3	4	5
13. If I encounter technical issues in creating course design content using an education-grade LLM agent, I believe I can handle it effectively.	1	2	3	4	5
14. I believe that the Education LLM Agency course will positively impact my course design abilities and instructional content creation.	1	2	3	4	5
15. The Education LLM Agent has enhanced my ability to solve the challenges of course content creation, as well as my critical thinking skills.	1	2	3	4	5
16. By using educational LLM agents, I have observed an improvement in course content creation standards.	1	2	3	4	5
17. The Education LLM Agent will positively influence my course content creation ability and teaching results.	1	2	3	4	5

Thank you again for taking the time to fill out this survey! Good luck with your work and happy life!
